# Concept mapping improves academic performance in problem solving questions in biochemistry subject

**DOI:** 10.12669/pjms.324.10432

**Published:** 2016

**Authors:** Mukhtiar Baig, Saba Tariq, Rehana Rehman, Sobia Ali, Zohair J Gazzaz

**Affiliations:** 1 Mukhtiar Baig, PhD, MHPE. Department of Clinical Biochemistry & Medical Education, Faculty of Medicine, Rabigh, King Abdulaziz University, Jeddah, KSA; 2 Saba Tariq, MPhil. Department of Pharmacology, University Medical & Dental College, Faisalabad, Pakistan; 3 Rehana Rehman, PhD. Department of Biological & Biomedical Sciences, Aga Khan University, Karachi, Pakistan; 4 Sobia Ali, MHPE. Department of Medical Education, Shalimar Medical and Dental College, Lahore. Pakistan; 5 Zohair J. Gazzaz, PhD. Department of Medicine, Faculty of Medicine, Rabigh, King Abdulaziz University, Jeddah, KSA

**Keywords:** Academic performance, Biochemistry, Concept map, MCQs

## Abstract

**Objective:**

To assess the effectiveness of concept mapping (CM) on the academic performance of medical students’ in problem-solving as well as in declarative knowledge questions and their perception regarding CM.

**Methods:**

The present analytical and questionnaire-based study was carried out at Bahria University Medical and Dental College (BUMDC), Karachi, Pakistan. In this analytical study, students were assessed with problem-solving questions (A-type MCQs), and declarative knowledge questions (short essay questions), and 50% of the questions were from the topics learned by CM. Students also filled a 10-item, 3-point Likert scale questionnaire about their perception regarding the effectiveness of the CM approach, and two open-ended questions were also asked.

**Results:**

There was a significant difference in the marks obtained in those problem-solving questions, which were learned by CM as compared to those topics which were taught by the traditional lectures (p<0.001), while no significant difference was observed in marks in declarative knowledge questions (p=0.704). Analysis of students’ perception regarding CM showed that majority of the students perceive that CM is a helpful technique and it is enjoyed by the students. In open-ended questions, the majority of the students commented positively about the effectiveness of CM.

**Conclusion:**

Our results indicate that CM improves academic performance in problem solving but not in declarative knowledge questions. Students’ perception about the effectiveness of CM was overwhelmingly positive.

## INTRODUCTION

In the medical field, there is an enormous explosion of knowledge, which is substantially increasing the breadth and depth of information, which medical students are supposed to learn and utilize. Therefore, in the present era of information technology, the greatest challenge for the medical educator is not only to overcome this information overload but also present this enormously expanding amount of information to the students in a more meaningful way that deepens their understanding. Many studies have described that CM is a better way of learning conceptual knowledge as compared to other conventional methods like reading, attending lectures and taking notes.^1^-^4^

Evidence in the literature suggests that CM is useful for better understanding of the subject, retaining information and improving students performance.^5^-^7^ Moreover, it is also very helpful in addressing the misconceptions of students, which normally cannot be detected by formal tests.^6^ Few studies have reported no significant improvement in students performance.^8^,^9^ A study in Pakistan reported that there was no significant effect on students performance, but it was liked by the students.^10^

Generally, medical students perceive Biochemistry as a difficult, volatile, content heavy subject in which many pathways and structures are involved. Therefore, there is always need to introduce innovative techniques, to make it more palatable and an easily understandable subject.

The present study was designed to assess the usefulness of CM technique as a learning approach for the entire class of the second year Medical Biochemistry during the carbohydrate metabolism course. We had the following two research questions:

1. What is the impact of using CM on students’ academic performance in problem solving and declarative knowledge questions?
2. How did the students’ find CM as a learning tool during the course?

## METHODS

This analytical and questionnaire-based study was carried out at Bahria University Medical and Dental College (BUMDC), Karachi, Pakistan in the year 2012. It is recommended that whenever a new method of learning is introduced in the classroom, its efficacy depends on students’ awareness of the method and their motivation about it.^11^ Therefore, to make them aware and motivate, a two-hour session for entire class was arranged to inform them about CM, its importance, and steps of its construction, based on the methods delineated by Novak & Canas (2007).^12^

In this novel experiment, we introduced CM technique as a learning tool for the entire class. In a whole month, during tutorial classes, students kept making made CM on different topics. This exercise was done to make them more familiar with the utilization and construction of CM. When all students had been acquainted with CM then in carbohydrate metabolism course, students were asked to construct CM on three topics of carbohydrate metabolism (glycolysis, gluconeogenesis & TCA cycle).

The students worked in pairs to complete concept map in the class, and the principal investigator provided them feedback on the quality, structural organization and integration of ideas. No traditional lectures were delivered on these three topics while other topics were taught in the traditional lecture format. Approval for this study was taken from the Biochemistry Department, and the study was conducted according to the principles of the Declaration of Helsinki, and the identity of the participants was not disclosed.

### Preparation of exam paper

After completion of carbohydrate metabolism, a test was arranged that comprised of problem-solving questions (A-type MCQs), and declarative knowledge questions (SEQs). Examination paper comprised of two sets of MCQs and SEQs; MCQs and SEQs from those topics which students learned by the CM and MCQs and SEQs from those topics which were taught by the traditional way. The quality and cognitive level of MCQs and SEQs for both sets were kept same to avoid the impact of quality of MCQs and SEQs on the performance of students and also to circumvent any biased with a particular method.

All students were asked to fill a 10-item, 3-point Likert scale questionnaire about their opinion on the effectiveness of the concept mapping approach. Two medical educationists reviewed the questionnaire for the format, clarity, style and suitability of the content.

### Analysis of data

We analyzed the data on SPSS 16 and compared the marks obtained in questions, which were taught by CM and traditional lectures by using Student’s t-test. The questionnaires of student perceptions regarding CM as an effective learning tool were evaluated by calculating the percentages of their response for each of the items. Students’ open-ended comments about advantages and disadvantages were analyzed for common themes.

## RESULTS

Students obtained significantly higher marks in problem solving questions (A-type MCQs) in the topics, which were learned by CM (for these topics no traditional lectures were delivered) compared to those topics in which lectures were delivered (p<0.001), while no significant difference was observed in marks in declarative knowledge questions (p=0.704). The total marks in topics learned by CM were also significantly higher from marks in topics taught by traditional lectures (p<0.01) (Table-I).

**Table-I:** Comparison of marks obtained in topics learned by concept mapping and taught by the traditional method.

Variables	Marks	Marks by traditional lecture (Total marks=15)	Marks by concept mapping (Total marks=15)	P-value
MCQs	30	11.01±0.20	12.23±0.20	0.001*
SEQs	30	11.06±0.18	11.16±0.20	0.704
Total	60	22.07±0.36	23.39±0.37	<0.01*

Results are shown as Mean±SEM,

* P value <0.05 was taken as significant.

Analyzes of students’ perception regarding CM showed that majority of the students perceive that CM is a helpful technique and enjoyed by the majority of the students’ (Table-II). We analyzed students’ responses to open-ended questions about the advantage/s and disadvantage/s of CM, for common themes. The majority of the students’ comments about the effectiveness of CM were overwhelmingly positive (Table-III).

**Table-II:** Responses of the students regarding the effectiveness of concept mapping.

Questions	Opinion N (%)
*CM helps in deep understanding of the topic*	
a) Completely	71(73.20)
b) Partly	13(13.40)
c) Not at all	13(13.40)
*CM helps in retaining information*	
a) Completely	64(65.98)
b) Partly	24(24.74)
c) Not at all	09(9.28)
*CM motivates to learn*	
a) Completely	57(58.76)
b) Partly	28(28.87)
c) Not at all	12(12.37)
*CM is a meaningful learning*	
a) Completely	66(68.04)
b) Partly	14(14.43)
c) Not at all	17(17.53)
*CM helps in correlating knowledge*	
a) Completely	43(44.33)
b) Partly	21(21.65)
c) Not at all	33(34.02)
*CM helps in self-assessment*	
a) Completely	53(54.64)
b) Partly	19(19.59)
c) Not at all	25(25.77)
*CM promotes active learning*	
a) Completely	65(67.01)
b) Partly	21(21.65)
c) Not at all	11(11.34)
*CM improved performance in MCQ test*	
a) Completely	47(48.45)
b) Partly	26(26.84)
c) Not at all	24(24.74)
*CM improved performance in SEQ test*	
a) Completely	36(37.11)
b) Partly	13(13.40)
c) Not at all	48(49.49)
*I enjoyed using concept map in this module*	
a) Completely	70(72.16)
b) Partly	14(14.43)
c) Not at all	13(13.40)

CM stands for concept mapping, N= number of respondents

**Table-III:** Results of open-ended questions arranged according to theme (What in your opinion is/are the main advantage/s & disadvantage/s of making concept maps?).

Comments	N(%)
*Advantage/s of making concept maps*	
1 Helpful in understanding the topic in depth	70 (72.16%)
2 Helpful in organizing thoughts	68 (70.10%)
3 Helpful in understanding all aspects of the topic	69 (71.13%)
4 Fast revision of the topic	69 (71.13%)
5 It makes learning meaningful	63 (64.94%)
6 It enhances the creativity	52 (53.61%)
*Disadvantage/s of making concept maps*	
1 It is a waste of time	27 (27.83%)
2 It didn’t help me in learning topic	19 (19.59%)
3 Difficult way of learning	22 (22.68%)
4 Very time consuming	45 (46.39%)
5 It is confusing	28 (28.87%)
6 It decreased my study timing	39 (40.21%)

N= number of respondents * Many students gave more than one comment.

## DISCUSSION

The present study found that the mean score of the students in problem-solving questions was significantly better on those topics which were studied by the CM. This finding is contradictory to several studies, which found no significant difference in students’ scores between the intervention and control groups.^5^-^7^ Our study results are similar to numerous studies.^8^-^9^,^13^

Interpretation of our study results is indicative of the fact that CM helped in the deeper understanding of the topics and because of that students’ obtained higher marks in problem-solving questions on those topics, which they learned via CM. CM is purely an active learning strategy that is very effective in retaining, recalling and applying knowledge and that is the reason they obtained better scores compared to those topics, which were taught by the traditional lecture format. Students’ scores in declarative knowledge questions were not different. It could be explained that SEQs in our exam were of low cognitive levels (eg. write down the reactions of galactose metabolism, enumerate the regulatory steps of glycolysis) and these need rote memorization and recall of information, while CM helps in conceptualization and deeper understanding of the topic, which is not needed in low cognitive level SEQs. The cognitive level strongly depends on the question and not necessarily on the format of the question.

In our study regarding the students’ perception about CM, the majority of students responded that CM is helpful in: deeper understanding about the topic, meaningful learning, self-assessment, correlating knowledge, retaining knowledge, and the majority of students admitted that it motivated them for learning, and they enjoyed using CM in this module. In open-ended questions about disadvantage/s of CM, many students mentioned that it is time-consuming and confusing while for advantage/s they mentioned that it is a way of active learning, helps in rapid revision, deeper understanding and organizing the thoughts.

A study in a Medical School in Australia found that 87% of the students agreed that CM was helpful in making links, 87% considered it enjoyable, 93% considered it a helpful tool in revision, 97% admitted that it provides valuable learning.^14^ Another study in Pakistan found that CM helped in understanding, assisting & enhancing thinking and learning in pharmacology, but students found CM time demanding and less helpful in attempting MCQs, and SEQs and majority of the students preferred it over the traditional method.^10^ Researchers also reported that students consider CM helpful to find out misconception and new connections, and a useful tool for visual learners.^15^ The researchers also described the results of qualitative evaluation of medical students learning with CM, and they reported that CM facilitates knowledge integration, critical thinking, and learning.^16^

Many of our participants responded that CM is a difficult task, confusing, and needs more time. These results are similar to several other studies, which have documented the similar perception of the many students.^10^,^16^,^17^ Brockevelt & Brydl-Andrews (2011) reported that many students found CM an arduous workload rather than a meaningful learning method and in their study, 81% of the students responded that CM did not assist them in understanding important course concepts.^17^

Educational researchers also addressed this issue by explaining that in the early stage of constructing CM, some students and teachers feel difficulty in constructing and using CM, because of the previous years’ rote-mode learning practices in their institutional settings.^12^ Later on, researchers described that it is a difficult task to facilitate students to shift from rote learning practices to a meaningful learning pattern. Furthermore, they explicated many students usually depend on rote memorization, while the concept map construction compels them to move out of their comfort zone and push them to search concepts and their interrelationships in a more innovative and holistic manner.^18^ It is suggested in the literature that in order to support students moving from rote to meaningful learning, the instructor should train them about the brain mechanisms and learning process by which they can learn maximally.^1^,^17^ Researchers in their meta-analysis of multiple disciplines, documented that CM was more useful for acquiring, retaining and applying knowledge, as compared to other conventional learning activities (such as lecture, reading, or class discussion).^4^ CM helps in deep and lifelong learning and it is recommended to adopt such technique that promote lifelong learning.^19^

There is a limitation to our study that it encompasses only second year Biochemistry MBBS students from only one medical college, limiting the generalization of our results to other subjects and other students. Therefore, it is suggested that further similar studies in other subjects are required to confirm these findings.

## CONCLUSION

Our results indicate that CM improves academic performance in problem-solving questions and students’ perception about the effectiveness of CM was overwhelmingly positive.
